# Cefminox, a Dual Agonist of Prostacyclin Receptor and Peroxisome Proliferator-Activated Receptor-Gamma Identified by Virtual Screening, Has Therapeutic Efficacy against Hypoxia-Induced Pulmonary Hypertension in Rats

**DOI:** 10.3389/fphar.2018.00134

**Published:** 2018-02-23

**Authors:** Jingwen Xia, Li Yang, Liang Dong, Mengjie Niu, Shengli Zhang, Zhiwei Yang, Gulinuer Wumaier, Ying Li, Xiaomin Wei, Yi Gong, Ning Zhu, Shengqing Li

**Affiliations:** ^1^Department of Pulmonary and Critical Care Medicine, Huashan Hospital, Fudan University, Shanghai, China; ^2^Department of Anesthesiology, Chongqing Medical University, Chongqing, China; ^3^Department of Gastroenterology Medicine, Xi'an Third Hospital, Xi'an, China; ^4^Department of Applied Physics, Xi'an Jiaotong University, Xi'an, China; ^5^Department of Respiratory Medicine, Shaanxi Provincial Second People's Hospital, Xi'an, China

**Keywords:** hypoxia-induced pulmonary hypertension, prostacyclin receptor, peroxisome proliferator-activated receptor-gamma, phosphatase and tensin homolog, cyclic adenosine monophosphate

## Abstract

Prostacyclin receptor (IP) and peroxisome proliferator-activated receptor-gamma (PPARγ) are both potential targets for treatment of pulmonary arterial hypertension (PAH). Expression of IP and PPARγ decreases in PAH, suggesting that screening of dual agonists of IP and PPARγ might be an efficient method for drug discovery. Virtual screening (VS) of potential IP–PPARγ dual-targeting agonists was performed in the ZINC database. Ten of the identified compounds were further screened, and cefminox was found to dramatically inhibit growth of PASMCs with no obvious cytotoxicity. Growth inhibition by cefminox was partially reversed by both the IP antagonist RO113842 and the PPARγ antagonist GW9662. Investigation of the underlying mechanisms of action demonstrated that cefminox inhibits the protein kinase B (Akt)/mammalian target of rapamycin (mTOR) signaling pathway through up-regulation of the expression of phosphatase and tensin homolog (PTEN, which is inhibited by GW9662), and enhances cyclic adenosine monophosphate (cAMP) production in PASMCs (which is inhibited by RO113842). In a rat model of hypoxia-induced pulmonary hypertension, cefminox displayed therapeutic efficacy not inferior to that of the prostacyclin analog iloprost or the PPARγ agonist rosiglitazone. Our results identified cefminox as a dual agonist of IP and PPARγ that significantly inhibits PASMC proliferation by up-regulation of PTEN and cAMP, suggesting that it has potential for treatment of PAH.

## Introduction

Currently approved treatments for pulmonary arterial hypertension (PAH) include calcium-channel blockers in vasoreactive patients, and drugs that target the endothelin, nitric oxide, and prostacyclin pathways in non-reactive patients (O'Connell et al., 2016). Despite considerable efforts in the development of new drugs in the past 20 years, the overall prognoses for patients with PAH remain poor (Velayati et al., 2016).

Prostacyclin (also known as prostaglandin I_2_) is produced in vascular endothelial cells and acts via the prostacyclin receptor (IP) of pulmonary artery smooth muscle cells (PASMCs) to cause vasodilation and inhibit PASMC proliferation (LeVarge, 2015). Prostacyclin production and IP expression are reduced in PAH (Saito et al., 2015), and analogs of prostacyclin are recommended as a pharmacotherapeutic option for patients with PAH in WHO Functional Class III/IV (Galie et al., 2016). The selective IP agonist oral selexipag has also displayed therapeutic effects, with acceptable tolerability in patients with PAH (Del Pozo et al., 2017; Honorato Pérez, 2017).

Peroxisome proliferator-activated receptor-gamma (PPARγ) is a nuclear receptor that functions as a transcription factor to regulate metabolism by binding to PPAR response elements (PPAREs) in the promoter regions of various target genes (Jung et al., 2012). Activation of PPARγ suppresses smooth muscle cell proliferation and migration by favorably regulating several potential pulmonary hypertension mediators (Rabinovitch, 2010; Sutliff et al., 2010). Treatment with the PPARγ agonist rosiglitazone prevents and reverses established hypoxia-induced pulmonary hypertension (HPH) by suppressing superoxide production and platelet-derived growth factor receptor-β signaling, and by increasing expression of the phosphatase and tensin homolog (PTEN) (Nisbet et al., 2010). Activation of PPARγ ameliorates monocrotaline-induced PASMC proliferation and vascular remodeling in a rat model of PAH by stimulation of expression of PTEN and inhibition of the phosphatidylinositide 3-kinase/protein kinase B(PI3K/Akt) pathway (Xie et al., 2015).

The existence of crosstalk between IP and PPARγ has been confirmed by results from several studies. In HEK293 cells, prostacyclin analogs activate PPARγ in the presence of IP; this activation is prevented by an IP antagonist (Falcetti et al., 2007). The anti-proliferative effects of prostacyclin analogs are also partially inhibited by PPARγ antagonists (Falcetti et al., 2007), suggesting that PPARγ is activated through IP by prostacyclin analogs, and that it contributes to the anti-proliferative effects. IP protein levels are lower in PASMCs from patients with idiopathic PAH (IPAH) than in unaffected individuals (Lai et al., 2008). Treatment of IP-deficient PASMCs with prostacyclin analogs inhibits cell growth in a cyclic adenosine monophosphate (cAMP)-independent, PPARγ-dependent manner (Falcetti et al., 2010). In IPAH PASMCs, anti-proliferative responses to IP analogs are insensitive to the presence or absence of IP, but are potentiated by a PPARγ agonist and inhibited by a PPARγ antagonist (GW9662) (Falcetti et al., 2010). The anti-proliferative effects of prostacyclin analogs are preserved in IPAH, despite IP down-regulation, by activation of PPARγ. These results suggest that the anti-proliferative effects of IP signaling can be complemented by PPARγ activation.

For this study, our hypothesis was that an agonist that targeted both IP and PPARγ would have better therapeutic efficacy than either an IP agonist or a PPARγ agonist. To identify potential dual agonists, receptor-based virtual screening (VS) was performed on the ZINC library (Irwin et al., 2012), utilizing two crystal structures of the PPARγ ligand-binding domain (LBD), as well as the homology model of IP. Potential interactions were further refined by molecular dynamics simulation, to clarify the mechanisms of action and important functional groups. Here, an IP and PPARγ dual agonist (cefminox) was selected for further investigation of its mechanism of action and therapeutic effects in a hypoxia-induced rat model of pulmonary hypertension (PH).

## Materials and methods

### Modeling studies

Two PPARγ-LBD crystal structures were retrieved from the Research Collaboratory for Structural Bioinformatics (RCSB) Protein DataBank (http://www.rcsb.org): an active form with binding to the full agonist rosiglitazone and coactivator peptide [entry code: 1FM6 (Gampe et al., 2000); defined herein as PPAR–full], and an active form with binding to the partial agonist (2S)-2-(biphenyl-4-yloxy)-3-phenylpropanoic acid (LRG) [entry code: 3B3K (Montanari et al., 2008); defined herein as PPAR–partial]. The coordinates for IP (accession number: NP_004387.1) were built with the MODELER module, with bovine rhodopsin (entry code: 1HZX; Teller et al., 2001) as a template (Stitham et al., 2003; Accelrys, 2011; Li et al., 2016; Xu et al., 2016). According to previously published protocols (Stitham et al., 2003; Pérez-Villa et al., 2015), each protein structure was first equilibrated by 100 ns explicit solvent molecular dynamics simulation using the GROMACS4.6.7 program (Pronk et al., 2013) and CHARMM27 force field (Brooks et al., 2009). Details of the molecular dynamics simulation setup were as previously described (Li et al., 2016; Xu et al., 2016). VS was performed on the ZINC compound database (http://zinc.docking.org/) (Irwin et al., 2012), using the cDocker program (Wu et al., 2003). The optimal orientations of compounds in relation to target proteins were probed on the basis of interactions with binding residues and geometrical matching qualities (Kilroy et al., 2012; Atanasov et al., 2013). Selected docked complexes were further refined by 100 ns molecular dynamics simulations, and binding free energies(Δ*G*_*bind*_) were evaluated by the molecular mechanics generalized born surface area method (g_mmpbsa) (Kumari et al., 2014), using previously described methods (Yang et al., 2013, 2016).

### Culture of primary rat PASMCs

Rat PASMCs were obtained via an explant method as described previously (Luo et al., 2013). PASMCs were grown in Dulbecco's modified Eagle's medium (DMEM) supplemented with 20% (v/v) fetal bovine serum, and verified by staining for smooth muscle α-actin at each passage (>95% of cells were positively stained). Cells were used at passage 3–6. In the cell-growth assay, PASMCs were exposed to normoxia or hypoxia, and treated with inhibitors or agonists as indicated. Cells in the normoxia group were maintained at 37°C in 21% O_2_, 74% N_2_, and 5% CO_2_ (HH·CP-01W humidified incubator; Shanghai Boxun Industry & Commerce, Shanghai, China). Cells in the hypoxia groups were separately cultured in 1% O_2_, 94% N_2_, and 5% CO_2_ for 48 h in a HERAcell 240 incubator (Heraeus, Kendro, Germany).

### Lentiviral infection

Lentiviral expression vectors (including Gipz-shPTEN) and packaging vectors (including pMD2.0G and psPAX) were purchased from Addgene (Cambridge, MA, USA). To prepare PTEN-knockdown viral particles, 293T cells were transfected with each viral vector and the packaging vectors (pMD2.0G and psPAX) using JetPEI transfection reagent (Qbiogene, Montreal, Canada), following the manufacturer's instructions. The medium was replaced 4 h after transfection, and cells were cultured for a further 36 h. Viral particles were harvested in culture medium, filtered through a 0.45 μm syringe filter, and combined with 8 μg/ml hexadimethrine bromide (Polybrene; Millipore, Boston, MA, USA), then added to PASMCs at 60% confluency, and incubated overnight. The culture medium was replaced with fresh complete growth medium, and cells were cultured for a further 24 h, then infected cells were selected with puromycin (8.0 μg/ml) for 36 h and used in further experiments.

### Cytotoxicity assay

PASMCs were seeded into 96-well plates at a density of 3,000 cells per well, and allowed to adhere in complete medium under normoxic conditions. The plated cells were treated with 0–500 μM cefminox for 48 h, after which the culture medium was removed. Cytotoxicity was evaluated with a CCK-8 cell proliferation assay kit (Beyotime, Shanghai, China). A total of 110 μl of DMEM containing CCK-8 [CCK8:DMEM(v/v) = 1:10] was added to each well, and cells were incubated for 4 h.Cell viability was determined by measuring absorbance at 450 nm using an Epoch Microplate Spectrophotometer (BioTek, Winooski, VT, USA).

### Quantitative RT-PCR

The mRNA levels of *PTEN* in PASMCs were determined by quantitative RT-PCR. Total RNA was extracted from PASMCs using TRIzol reagent (Invitrogen, Life Technologies, Carlsbad, CA, USA) according to the manufacturer's protocol. Reverse transcription was performed using SuperScript II reverse transcriptase (Life Technologies). cDNA was amplified and detected using SYBR Premix Ex Taq (TaKaRa, Dalian, China). *ACTB* (encoding β-actin) was used as an internal loading control. The PCR primers were as follows: 5′-GATCATTGCTCCTCCTGAGC-3′ and 5′-ACTCCTGCTTGCTGATCCAC-3′ for *ACTB*, and 5′-CGACGGGAAGACAAGTTCAT-3′ and 5′-AGGTTTCCTCTGGTCCTGGT-3′ for *PTEN*.

### Western-blotting analysis

Total lysates of harvested lung tissue and cultured PASMCs were obtained. Lung homogenates were prepared in RIPA lysis buffer (Beyotime, Jiangsu, China). The protease inhibitor phenylmethylsulfonyl fluoride (final concentration, 1 mM) was added to the buffer immediately prior to use. Equivalent amounts of protein were separated by electrophoresis in SDS-polyacrylamide gels and transferred to 0.22 μm pore-size nitrocellulose membranes (Millipore). Primary antibodies against phospho-Akt (S473, used at 1:1,000 dilution), Akt (1:1,000), phospho-mTOR (mammalian target of rapamycin; S2481, 1:1,000), mTOR (1:1,000), and IP (1:1,000) were purchased from Cell Signaling Technology (Danvers, MA, USA). Primary antibodies against PTEN (1:1,000) and β-actin (1:1,000) were purchased from Abcam (Cambridge, UK). A primary antibody against PPARγ (1:1,000) was purchased from Santa Cruz Biotechnology (Dallas, TX, USA). Membranes were incubated with primary antibodies overnight at 4°C and then (after washing) with horseradish peroxidase-conjugated secondary antibodies for 1 h at room temperature. Chemiluminescence signals were detected using a WesternBright ECL kit (Advansta, Menlo Park, CA, USA).

### Intracellular cAMP measurement

PASMCs were grown to 70–80% confluence in 6-well plates, then starved in DMEM containing 0.1% FBS for 48 h, before being stimulated with agonists and/or antagonists for 30 min in medium containing 10% FBS. cAMP was extracted and measured using a competitive enzyme immunoassay kit (Cyclic AMP ACE EIA kit, Cayman Chemical, Ann Arbor, MI, USA), according to the manufacturer's instructions. Protein concentration was determined using the Bradford assay (Bio-Rad Laboratories, Hemel Hempstead, UK).

### Hypoxia-induced rat model of PH

Adult male Sprague Dawley (SD) rats weighing 150–200 g were purchased from the Laboratory Animal Center, Fourth Military Medical University (Xi'an, China). All protocols and surgical procedures were approved by the Fourth Military Medical University Veterinary Medicine Animal Care and Use Committee.

Animals were randomly allocated into six groups (*n* = 8 per group): (1) normoxia; (2) chronic hypoxia; (3–6) chronic hypoxia treated with (3) iloprost (1.5 μg/kg via 2 ml inhalation, six times daily); (4) rosiglitazone (10 mg/kg daily, gavage administration); (5) low dose cefminox (160 mg/kg daily, tail intravenous injection); (6) high dose cefminox (320 mg/kg daily, tail intravenous injection). Animals in normoxic groups were housed at ambient barometric pressure for 28 days (~718 mmHg, PO_2_~150.6 mmHg). Animals in hypoxic groups were housed in a hypobaric hypoxia chamber depressurized to 380 mmHg (PO_2_ reduced to ~79.6 mmHg, accordingly) 8 h per day for 28 days. All animals were raised in a 12 h light−12 h dark cycle, and supplied with food and water *ad libitum*. Room temperature was maintained at 25°C, and the bedding was changed once a week.

### Hemodynamic analysis and tissue preparation

After 28 days, rats were fasted overnight and anesthetized with 20% ethyl carbamate (4 ml/kg by intraperitoneal injection). In each anesthetized animal, the right jugular vein was carefully isolated, a specially shaped catheter linked to the PowerLab system (AD Instruments, Bella Vista, NSW, Australia) was inserted into the right ventricle (RV) via this vein, and right ventricular systolic pressure (RVP) was recorded. Then, sternotomy was performed and the rat was perfused with 4% paraformaldehyde. Both lungs and the heart were harvested. The RV and the left ventricle plus septum (LV+S) were weighed, and the RV:LV+S weight ratio was determined, to give an indication of right ventricular hypertrophy. Next, the lower lobe of the right lung was sectioned into slices 4 mm thick, which were soaked in 10% formalin solution (pH = 7.4). Other tissues were kept at −80°C until required.

### Hematoxylin and eosin staining

Fixed lungs were sliced in the mid-sagittal plane, embedded in paraffin, and cut into ~5 μm sections with a microtome. The sections were placed on glass slides, stained with hematoxylin and eosin (HE) for morphological analysis, and visualized with an Olympus BX41 microscope (Tokyo, Japan).

### Statistical analysis

Statistical significance was assessed by comparing mean (±*SD*) values with Student's *t*-test for independent groups. *P* ≤ 0.05 was considered statistically significant.

## Results

### Screening of IP and PPARγ dual-agonist cefminox

**Figure 1:**
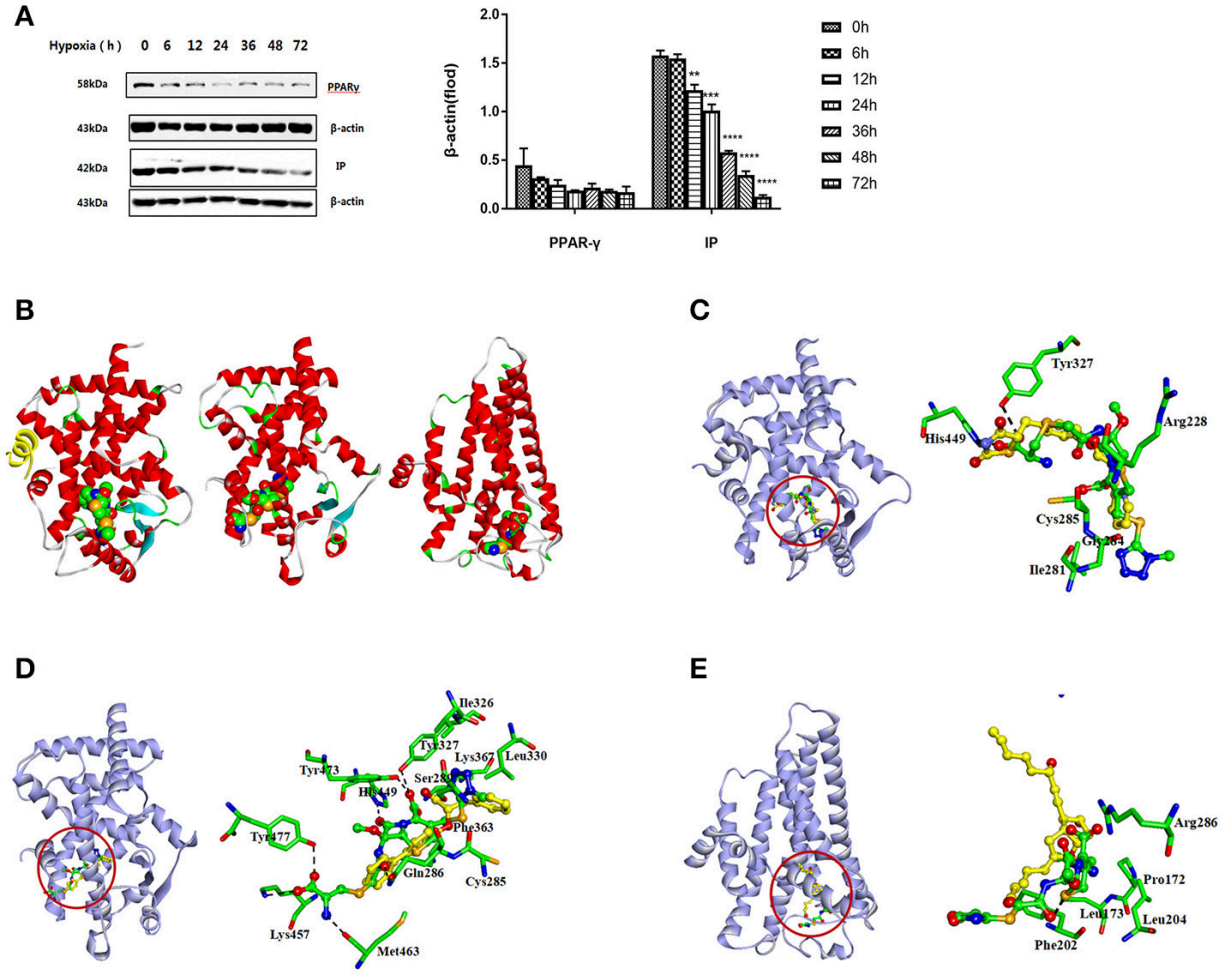
Screening of IP and PPARγ dual agonists. **(A)** Primary rat pulmonary artery smooth muscle cells (PASMCs) were cultured under normoxic or hypoxic conditions, and the levels of prostacyclin receptor (IP) and peroxisome proliferator-activated receptor-gamma (PPARγ) proteins were detected by western blotting at the indicated time points. All protein levels were measured by densitometry and normalized to that of β-actin. Each bar represents the mean ± *SD*. For statistical significance, ^**^p < 0.01, ^***^p < 0.001, and ^****^p < 0.0001, compared with control values. **(B)** Predicted orientations of cefminox within PPAR–full (Protein DataBank (PDB) ID: 1FM6, Chain A), PPAR–partial (PDB ID: 3B3K, Chain A), and IP. PPAR–full represents the fully active form of PPARγ ligand-binding domain (LBD) with coactivator peptide (yellow ribbon). PPAR–partial represents the partially active form of PPARγ LBD. The O, N, and C atoms are colored in red, blue, and green, respectively. **(C–E)** Equilibrium structures of cefminox interacting with **(C)** PPAR–full, **(D)** PPAR–partial, and **(E)** IP. Key residues and compounds are represented by stick and ball-and-stick models, respectively. C atoms are colored yellow for rosiglitazone in PPAR–full, (2S)-2-(biphenyl-4-yloxy)-3-phenylpropanoic acid (LRG) in PPAR–partial, and prostacyclin in IP. Important hydrogen bonds are shown by dashed black lines.

**Figure 2:**
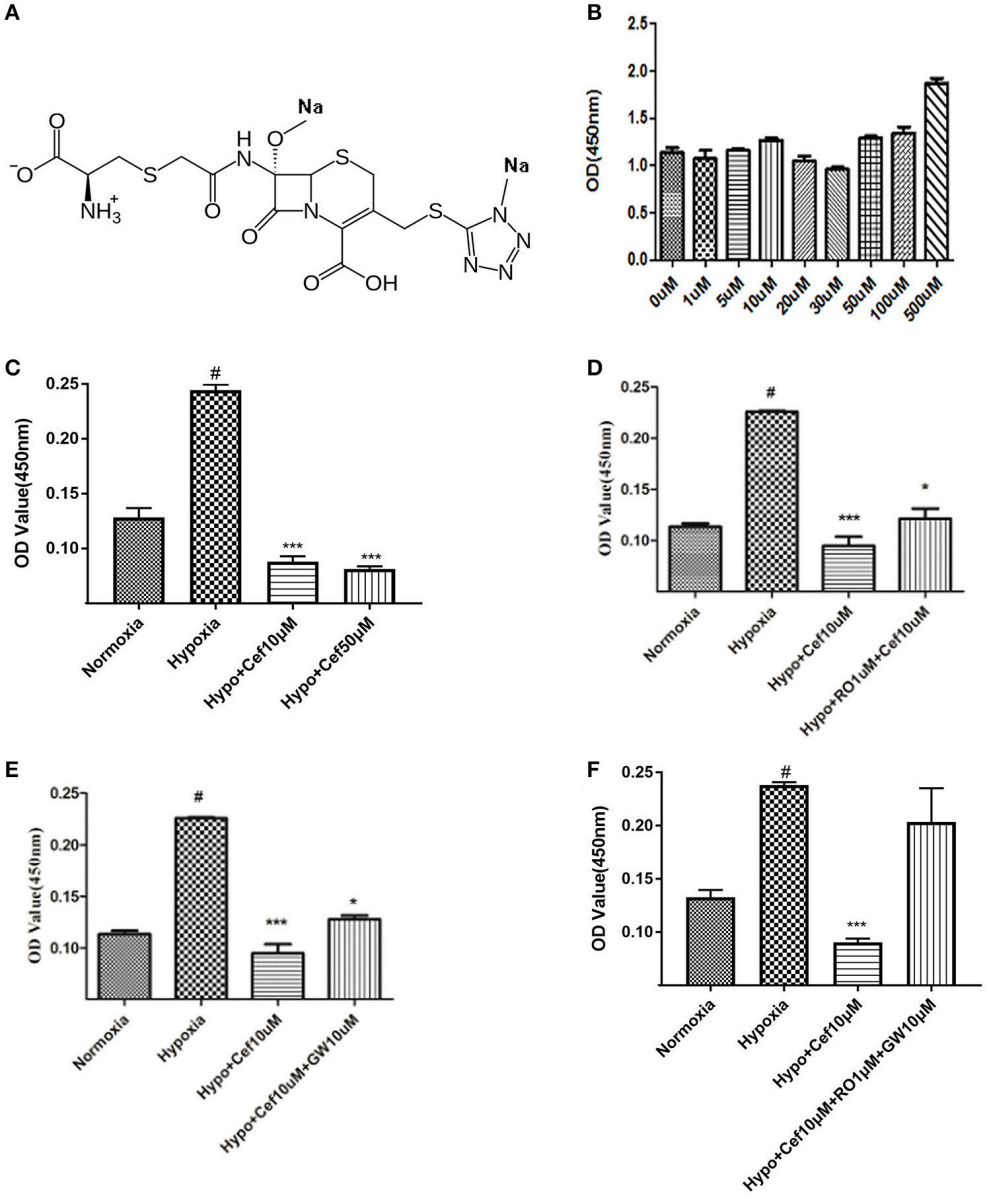
Cefminox inhibits cell growth under hypoxic conditions. **(A)** Molecular structure of cefminox. **(B)** Primary pulmonary artery smooth muscle cells (PASMCs) were cultured under normoxic conditions and treated with different doses of cefminox, then cytotoxicity was assayed. **(C–E)** Primary PASMCs were cultured under normoxic or hypoxic (Hypo) conditions and treated for 48 h, then cell growth was assayed. **(C)** PASMCs were treated with different doses of cefminox (Cef). **(D)** PASMCs were treated with 10 μM cefminox and 1 μM RO113842 (RO) prostacyclin-receptor antagonist. **(E)** PASMCs were treated with 10 μM cefminox and 10 μM GW9662 (GW) peroxisome proliferator-activated receptor-gamma antagonist. **(F)** PASMCs were treated with 10 μM cefminox and 10 μM GW9662 (GW) combined with 1 μM RO113842 (RO). Data from three independent experiments are shown as means ± *SD*s. For statistical significance, ^*^^#^*p* < 0.05 and ^***^p < 0.001, compared with control values.

Primary rat PASMCs were cultured under hypoxic conditions, leading to time-dependent reduction in the protein levels of IP and PPARγ, compared with cells cultured under normoxic conditions (Figure 1A). Evidence of crosstalk between IP and PPARγ signaling in PAH suggested that screening of dual agonists of IP and PPARγ might be an efficient method for therapeutic drug discovery. Compounds with dual binding capacities for PPARγ and IP were identified by VS and ranked by combined cDocker interaction energies (*E*_*int*_); the top ten compounds are listed in Table S1. All these compounds were screened for cytotoxicity and the potential of anti-proliferation. *In vitro* assays determined that cefminox was not cytotoxic, and that it had anti-proliferative properties in primary cultured PASMCs under hypoxic conditions (Figure 2). Interaction energies for cefminox with PPAR–full, PPAR–partial, and IP structures determined with cDocker are shown in Table 1. These binding properties were further studied by explicit solvent molecular dynamics simulations, and each system (details in Figure 1B) sufficiently equilibrated after about 80 ns, verified by the time evolution of various structural parameters (Figure S1) (Grossfield and Zuckerman, 2009).

**Table 1:** Comparison of cDocker interaction energies (*E*_*int*_) for interactions between specific compounds and proteins^a^.

**Compound^b^**	**Full**	**Partial**	**PPAR_γ_^c^**	**IP**	**PPAR_γ_+ IP**	**Application**
Cefminox	−65.07	−70.02	−67.54	−63.70	−131.24	Antibiotics
RSG	−58.15	−49.87	−54.01	−42.39	−96.40	Control for Full
LRG	−50.04	−66.63	−58.34	−38.79	−97.13	Control for Partial
PGI_2_	−51.64	−60.91	−56.27	−51.25	−107.52	Control for IP

a *All values are given in kcal·mol^−1^, obtained by the cDocker module (Discovery Studio 3.1)*.

b *RSG, rosiglitazone; LRG, (2S)-2-(biphenyl-4-yloxy)-3-phenylpropanoic acid; PGI_2_, prostacyclin*.

c *Averages of the first two groups*.

The predicted binding of cefminox to PPAR–full was similar to that of the full agonist rosiglitazone, which stabilizes the AF2 surface that is associated with a common horseshoe conformation centered about H3 (Figures 1B,C; Farce et al., 2009). The free terminal carboxyl group of cefminox exhibited strong hydrogen-bonding interactions with PPAR–full residues His449 and Tyr327. Modest hydrophobic interactions were predicted between the hydrophobic portions of residues (Arg228, Cys285, Ile281, Gly284) and the thiazine (tetrazole) ring of cefminox (Figure 1C). The predicted binding of cefminox with PPAR–partial resembled that of the partial agonist LRG, which binds in the ligand-binding pocket in a hydrophobic manner (including residue Cys285) and stabilizes the β-sheet region (residues Ser289/Ser342) through hydrogen bonding (Figure 1B,D; Farce et al., 2009; Kroker and Bruning, 2015). The binding free energies (Δ*G*_*bind*_) of cefminox with PPAR–full and PPAR–partial were −40.60 ± 4.24 and −52.49 ± 7.25 kcal·mol^−1^, which were greater than those of rosiglitazone in PPAR–full (−40.04 kcal·mol^−1^) and LRG in PPAR–partial (−43.23 kcal·mol^−1^; Table 2). Van der Waals components (Δ*E*_*vdw*_ + Δ*G*_*sur*_) primarily drove the binding processes, with values for cefminox of −61.16 kcal·mol^−1^ for PPAR–full and −58.70 kcal·mol^−1^ for PPAR–partial, which were consistent with the values for rosiglitazone (−54.15 kcal·mol^−1^) and LRG (−47.86 kcal·mol^−1^; Table 2).

**Table 2:** Predicted binding free energies (Δ*G*_*bind*_) and their components for specific complexes of compounds and proteins^a^.

**Complex^b^**	**Δ*E_*ele*_***	**Δ*E_*vdw*_***	**Δ*G_*sur*_***	**Δ*G_*GB*_***	**Δ*G_*bind*_***
Cef-**Full**	−14.13 ± 8.26	−54.06 ± 3.92	−7.10 ± 0.37	34.69 ± 7.35	−40.60 ± 4.24
Cef-**Partial**	−91.56 ± 34.27	−51.04 ± 4.55	−7.66 ± 0.36	97.77 ± 30.53	−52.49 ± 7.25
Cef-**IP**	−141.01 ± 18.28	−49.87 ± 3.59	−7.19 ± 0.27	138.38 ± 15.08	−59.69 ± 4.91
RSG-**Full**	−14.46 ± 3.86	−47.86 ± 3.00	−6.29 ± 0.28	28.57 ± 2.41	−40.04 ± 3.82
LRG-**Partial**	−26.02 ± 13.98	−41.59 ± 3.31	−6.27 ± 0.25	30.65 ± 10.79	−43.23 ± 5.09
PGI_2_-**IP**	−146.00 ± 17.32	−51.47 ± 4.51	−6.75 ± 0.54	169.33 ± 14.91	−34.89 ± 5.56

a *All values are given in kcal·mol^−1^, and following “±” are their standard deviations (SD)*.

b *RSG, rosiglitazone; LRG, (2S)-2-(biphenyl-4-yloxy)-3-phenylpropanoic acid; PGI_2_, prostacyclin*.

The predicted binding of cefminox with IP resembled that of prostacyclin, which is the natural ligand of IP (Figures 1B,E; Haché et al., 2003). The thiazine ring of cefminox exhibited a strong hydrogen-bonding interaction with residue Phe202, and the thiazine and tetrazole rings of cefminox had stable hydrophobic interactions with the hydrophobic portions of residues Pro172, Leu173, Phe202, Leu204, and Arg286 (Figure 1E), which was similar to the previous molecular modeling of prostacyclin–IP (Stitham et al., 2003). The binding free energy of cefminox with IP (−59.69 kcal·mol^−1^) was higher than that of prostacyclin (−34.89 kcal·mol^−1^; Table 2). Van der Waals components were the chief driving force for the binding process, with a value of −57.06 kcal·mol^−1^, compared with −58.22 kcal·mol^−1^ for prostacyclin. As cefminox has binding affinities with PPAR–full, PPAR–partial, and IP that are comparable to those of rosiglitazone, LRG, and prostacyclin, it has potential as a dual-targeting agonist with possible therapeutic effects for PAH.

### Cefminox inhibited PASMC growth

For the compounds identified as potential IP and PPARγ dual agonists (Table S1), a cytotoxicity assay was employed to exclude those with notable cytotoxicity, and to determine safe dose ranges for further cell-growth studies. Cefminox had no observable cytotoxicity to cultured primary PASMCs, even at a dose of 500 μM (Figures 2A,B). In studies of PASMC growth, hypoxia dramatically increased cell proliferation, and this effect was significantly inhibited by cefminox in a dose-dependent manner (Figure 2C). Inhibition of hypoxia-induced proliferation by cefminox was partially reversed by the IP antagonist RO113842 (Figure 2D) and by the PPARγ antagonist GW9662 (Figure 2E), and was almost totally reversed by the combination of RO113842 and GW9662 (Figure 2F). These results suggested that cefminox inhibits PASMC proliferation through enhancement of both IP and PPARγ activities.

### Cefminox enhanced PTEN expression in PASMCs

**Figure 3:**
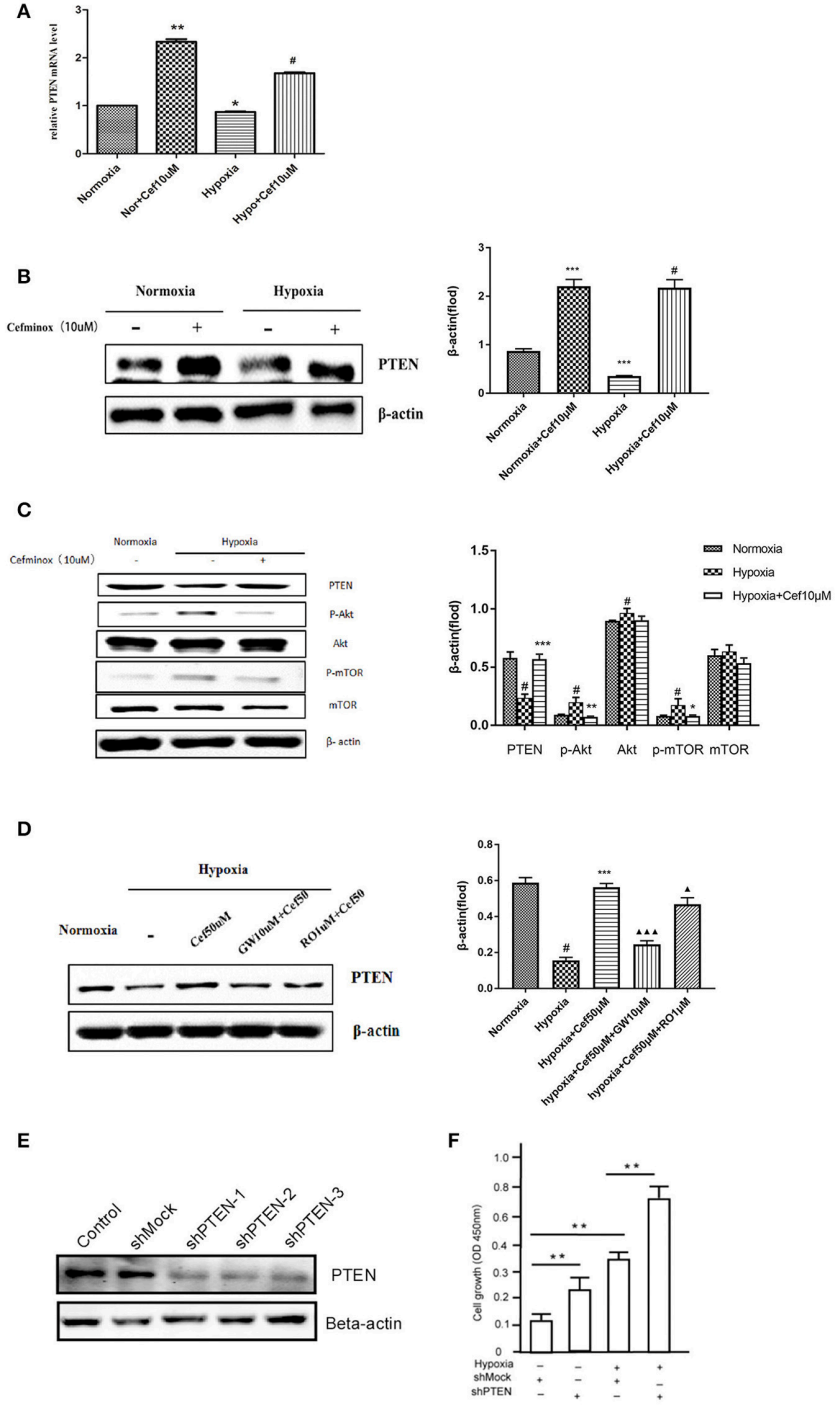
Cefminox inhibits Akt/mTOR signaling by elevating cellular PTEN expression. Primary pulmonary artery smooth muscle cells (PASMCs) were cultured under normoxic or hypoxic (Hypo) conditions and treated with 10 μM cefminox (Cef), to determine **(A)** *PTEN* mRNA levels by quantitative RT-PCR, **(B)** PTEN protein level by western blotting, and **(C)** activity of the Akt/mTOR signaling pathway. **(D)** Primary PASMCs were cultured under normoxic or hypoxic conditions and treated with 10 μM cefminox, 1 μM RO113842 (RO) prostacyclin-receptor antagonist, and 10 μM GW9662 (GW) peroxisome proliferator-activated receptor-gamma antagonist, and PTEN protein levels were determined by western blotting. **(E)** PTEN expression in PASMCs was knocked down with different short hairpin RNAs (shRNAs), and the knockdown efficiency was determined by western blotting. **(F)** Growth of PASMCs was assayed following infection with shMock or shPTEN-1 encoding lentiviruses under either normoxia condition or hypoxia condition. Data from three independent experiments are shown as means ± *SD*s. All protein levels were measured by densitometry and normalized to that of β-actin. For statistical significance, ^Δ*#^*p* < 0.05, ^**^p < 0.01, and ^ΔΔΔ***^p < 0.001, compared with control values.

Activation of PPARγ inhibits cell proliferation through up-regulation of expression of the tumor suppressor PTEN in human promyeloid leukemia cells (Lee et al., 2007), non-small-cell lung cancer (A549) cells (Lee et al., 2006), and PASMCs by inhibition of the PI3K/Akt pathway (Xie et al., 2015). In our experiments, hypoxia resulted in decreased *PTEN* mRNA expression in PASMCs, compared with normoxia (Figure 3A). As an agonist of PPARγ, cefminox increased *PTEN* mRNA levels (Figure 3A) and PTEN protein levels (Figure 3B) in both normoxic and hypoxic conditions. The down-regulation of PTEN that was induced by hypoxia caused activation of downstream Akt/mTOR signaling, which was demonstrated by elevated levels of phosphorylation of Akt and mTOR (Figure 3C). Cefminox inhibited the Akt/mTOR signaling pathway through elevation of PTEN protein levels (Figure 3C). Up-regulation of PTEN by cefminox under hypoxic conditions was reversed by the PPARγ antagonist GW9662, but not by the IP antagonist RO113842 (Figure 3D). Activation of Akt/mTOR signaling promotes cell proliferation and cell survival, and to confirm that PTEN inhibits the growth of PASMCs, the effects of *PTEN* knockdown in PASMCs were determined by cell-proliferation assays, which showed that cells with *PTEN* knockdown grew more rapidly than control cells either in normoxia condition or in hypoxia condition (Figures 3E,F). These results indicated that cefminox inhibits cell growth by up-regulating PTEN expression and inhibiting Akt/mTOR signaling in PASMCs cultured under hypoxic conditions.

### Cefminox enhanced cAMP production in PASMCs

**Figure 4:**
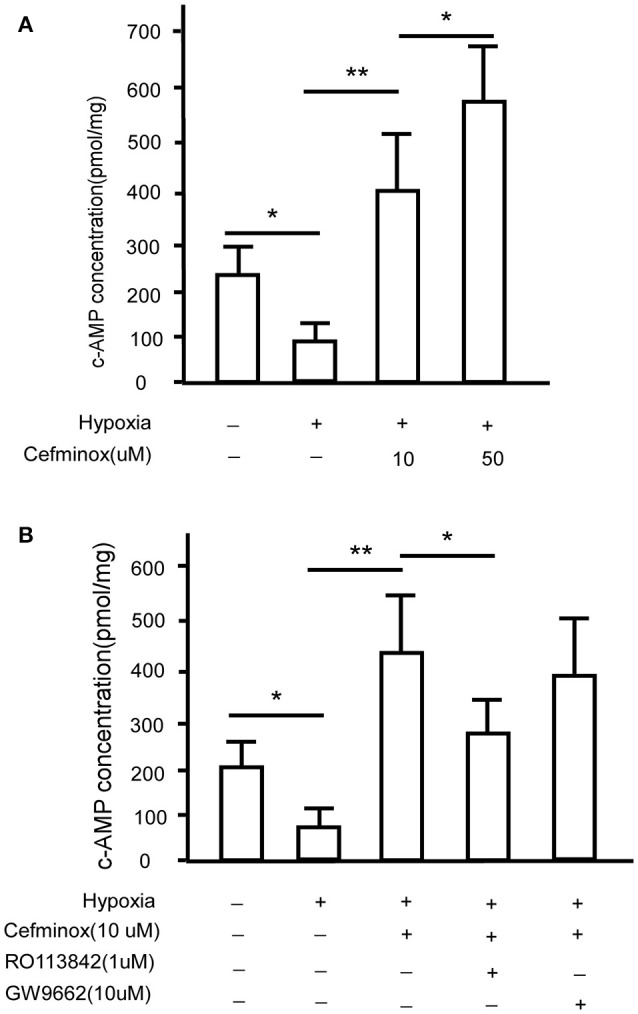
Cefminox up-regulates cellular cAMP production. Primary pulmonary artery smooth muscle cells (PASMCs) were cultured under normoxic or hypoxic conditions and treated with **(A)**10 or 50 μM cefminox, or **(B)**10 μM cefminox, 1 μM RO113842, and 10 μM GW9662, and cAMP concentrations were assayed. Data from three independent experiments are shown as means ± *SD*s. For statistical significance, ^*^p < 0.05, ^**^p < 0.01 compared with control values.

Prostacyclin is produced in vascular endothelial cells through the action of the enzyme prostacyclin synthase (Vane and Corin, 2003), and acts via IP to convert ATP to cAMP (Gomberg-Maitland and Olschewski, 2008), causing vasodilation and inhibition of smooth muscle cell proliferation and platelet aggregation (Humbert and Ghofrani, 2016; Del Pozo et al., 2017). To determine whether IP is activated by cefminox, PASMCs were cultured under hypoxic conditions, which induced down-regulation of levels of cAMP; in these conditions, addition of cefminox dramatically increased cAMP production in a dose-dependent manner (Figure 4A). The cefminox-induced up-regulation of cAMP in PASMCs was inhibited by the IP antagonist RO113842, but not by the PPARγ antagonist GW9662 (Figure 4B). These data indicated that cefminox inhibits PASMC proliferation by activation of IP and up-regulation of cAMP production.

### Therapeutic effects of cefminox in a rat model of HPH

**Figure 5:**
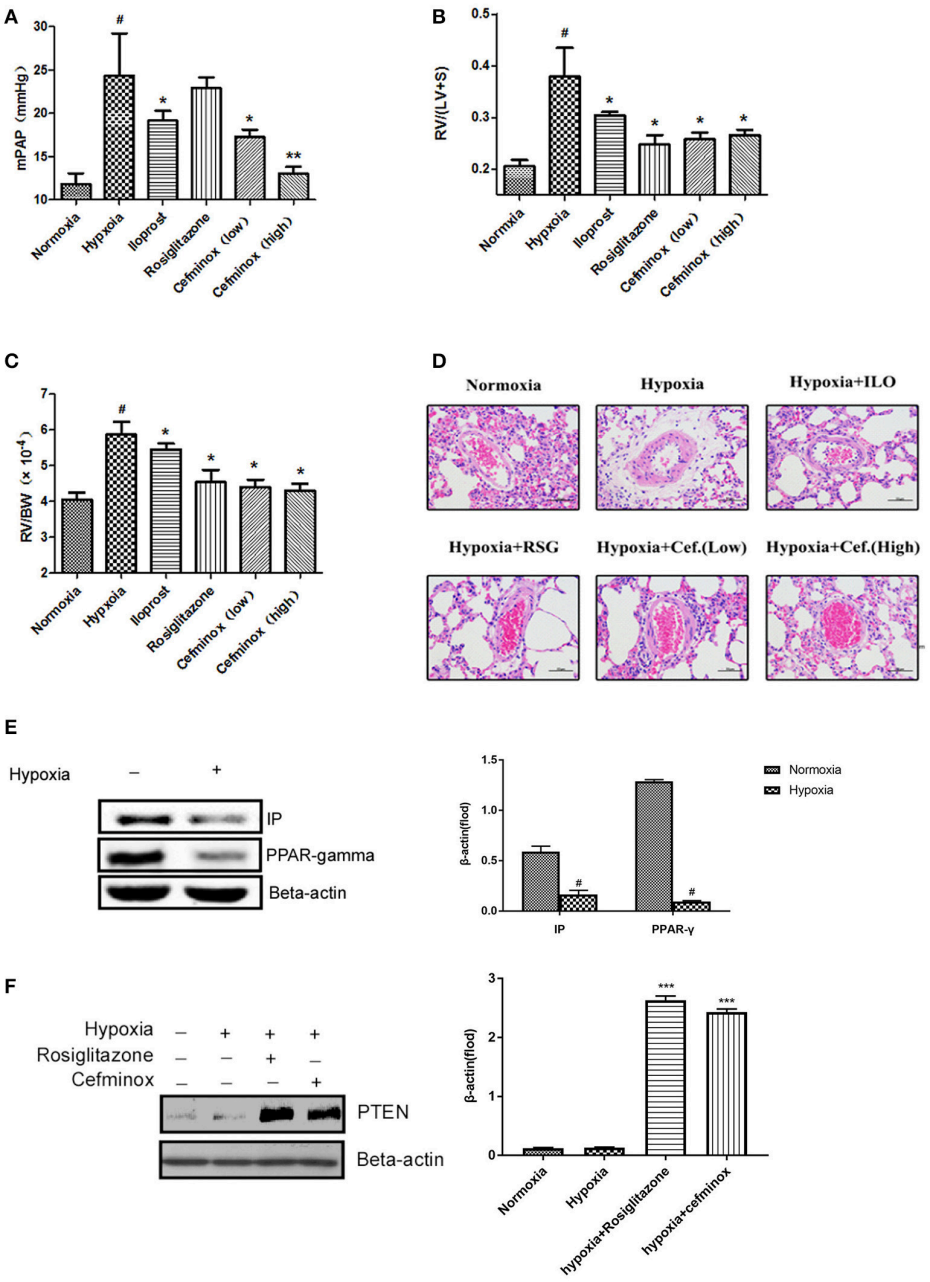
Cefminox significantly reverses hypoxia-induced pulmonary hypertension. A rat model of hypoxia-induced pulmonary hypertension (HPH) was generated (*n* = 8 animals per group). Experimental groups were treated with different doses of cefminox, iloprost, and rosiglitazone, with subsequent calculation of **(A)** the mean pulmonary artery pressure (mPAP), **(B)** the weight ratio of the right ventricle (RV) to the left ventricle plus septum (LV+S), and **(C)** the weight ratio of the RV to body weight (BW). **(D)** Hematoxylin and eosin (HE) staining of paraffin-fixed lung sections for morphological analysis of pulmonary arteries. **(E,F)** Western blotting of protein extracts from lung tissues, probed with the indicated antibodies. Data shown are means ± *SD*s. All protein levels were measured by densitometry and normalized to that of β-actin. For statistical significance, ^*^^#^*p* < 0.05, ^**^p < 0.01, and ^***^p < 0.001, compared with control values.

In a rat model of HPH, cefminox significantly decreased mean pulmonary artery pressure (mPAP) in a dose-dependent manner (Figure 5A), and inhibited RV remodeling, decreasing both the RV:(LV+S) (Figure 5B) and RV:body weight (BW) ratios (Figure 5C) compared with those of rats exposed to hypoxia alone. The therapeutic effect of cefminox was not inferior to that of the prostacyclin analog iloprost or the PPARγ agonist rosiglitazone. Morphological analysis revealed that cefminox significantly reversed hypoxia-induced pulmonary artery remodeling by decreasing the number of smooth muscle cells in the media (Figure 5D). In this rat HPH model, expression of PPARγ and IP in lung tissues decreased dramatically after 4 weeks of hypoxia, compared with the normoxia group (Figure 5E). In the HPH groups with either rosiglitazone or cefminox treatment, expression of PTEN increased compared with that in the normoxia or hypoxia (no treatment) groups (Figure 5F). The results of these animal studies indicated that cefminox has therapeutic efficacy to HPH, and that it is not inferior to either iloprost or rosiglitazone.

Overall, our results indicated that hypoxia induces down-regulation of expression of both IP and PPARγ *in vitro* and *in vivo*. As a dual agonist of IP and PPARγ, cefminox significantly inhibited growth of PASMCs through up-regulation of PTEN expression and cAMP production, and reversed remodeling of the pulmonary artery and RV in a rat HPH model. Cefminox, therefore, has potential as a novel treatment for PAH.

## Discussion

Expression of IP and PPARγ decreases in the pulmonary arteries of patients with PAH, compared with unaffected individuals (Ameshima et al., 2003; Falcetti et al., 2010), and this effect was also demonstrated in our rat HPH model. Analogs of prostacyclin and agonists of IP have been used successfully in the treatment of patients with PAH (Leuchte et al., 2003; Baker et al., 2017). Activation of PPARγ exerts anti-proliferative, anti-thrombotic, and vasodilatory effects on the vasculature in PAH, and agonists of PPARγ have efficacy in the treatment of animal models of PAH (Green et al., 2011; Liu et al., 2014). In this study, we screened the ZINC compound database and identified cefminox, a dual agonist of IP and PPARγ, which significantly inhibited PASMC growth through up-regulation of PTEN expression and cAMP production. Cefminox had therapeutic efficacy in the rat HPH model that was not inferior to that of iloprost (a prostacyclin analog) or rosiglitazone (a PPARγ agonist). Our results suggest that dual agonists of IP and PPARγ can be a novel class of drug for the treatment of PAH.

Cefminox is an antibiotic, and its long-term use carries the risk of causing dysbiosis of respiratory tract flora. The standard dose of cefminox for the treatment of pulmonary infection is 6 g per day (Odagiri et al., 1985), and the MIC80 (minimum concentration for 80% inhibition) values of cefminox for *Haemophilus influenzae, Escherichia coli, Streptococcus pyogenes*, and *Staphylococcus aureus* range from 1.56 to 12.5 mg/ml (Iwai et al., 1985). Our experiments involved doses of 160–320 mg/kg per day for the treatment of HPH rats, and further study would be necessary to ensure that these doses of cefminox have no detrimental influence on the respiratory tract flora, before cefminox could be used for the long-term treatment of PAH. It might be possible to modify the molecular structure of cefminox or change the route of administration, to retain its therapeutic efficacy for PAH while minimizing the antibiotic activity. Alternatively, new molecules could be designed and synthesized to target both IP and PPARγ.

In conclusion, VS of the ZINC database identified the IP and PPARγ dual agonist cefminox, which inhibited growth of PASMCs through activation of PPARγ signaling by up-regulation of expression of PTEN, as well as enhancement of IP signaling by up-regulation of cAMP production. In a rat HPH model, cefminox displayed therapeutic efficacy not inferior to that of the prostacyclin analog iloprost or the PPARγ agonist rosiglitazone. Cefminox, as a dual agonist of IP and PPARγ, has potential as a novel therapeutic drug for PAH.

## Summary

Cefminox, a dual agonist of IP and PPARγ, inhibited PASMC growth by up-regulation of PTEN expression and cAMP production, and displayed therapeutic efficacy not inferior to iloprost or rosiglitazone in a HPH rat model.

## Ethics statement

This study was carried out in accordance with the recommendations of Xijing Hospital Ethics Committee.The protocol was approved by the Xijing Hospital Ethics Committee.

## Author contributions

SL is responsible for the experiment design and organization, manuscript preparation and the proof correction. All authors listed have made a substantial, direct and intellectual contribution to the work, and approved it for publication.

### Conflict of interest statement

The authors declare that the research was conducted in the absence of any commercial or financial relationships that could be construed as a potential conflict of interest.

## Abbreviations

HPH: hypoxia-induced pulmonary hypertension
IP: prostacyclin receptor
PPARγ: peroxisome proliferator-activated receptor-gamma
PTEN: phosphatase and tensin homolog
PASMC: pulmonary artery smooth muscle cell.
