# Supplementary material for: Cefminox, a Dual Agonist of Prostacyclin Receptor and Peroxisome Proliferator-Activated Receptor-Gamma Identified by Virtual Screening, Has Therapeutic Efficacy against Hypoxia-Induced Pulmonary Hypertension in Rats
Source: Front Pharmacol. 2018 Feb 23;9:134. doi: 10.3389/fphar.2018.00134 (PMC5829529; doi:10.3389/fphar.2018.00134)
Supplement: Supplementary file 1 [file Table1.DOCX]

| **Table S1.** Screening results of top ten compounds according to the cDocker interaction energies (*E_int_*) between compounds and proteins*^a^* | | | | |
| --- | --- | --- | --- | --- |
| **No.** | **ID***^b^* | **PPAR_γ_***^c^* | **IP** | **PPAR_γ_ + IP** |
| 1 | Gusperimus (INN) | -84.87 | -78.83 | -163.70 |
| 2 | Nonaethylene Glycol | -72.91 | -65.85 | -138.76 |
| 3 | Miltefosine | -68.73 | -68.48 | -137.21 |
| 4 | Hexoprenaline | -64.75 | -71.99 | -136.74 |
| 5 | ZINC85569445 | -68.58 | -68.15 | -136.73 |
| 6 | S-(p-nitrobenzyl)glutathione | -72.32 | -62.80 | -135.13 |
| 7 | Imatinib | -64.92 | -69.25 | -134.17 |
| 8 | S-hexylglutathione | -69.24 | -64.42 | -133.66 |
| 9 | ZINC85628393 | -65.45 | -65.97 | -131.42 |
| **10** | **Cefminox Sodium** | **-67.54** | **-63.70** | **-131.24** |
| Control for **Full** | RSG | -56.27 | -51.25 | -107.52 |
| Control for **Partial** | LRG | -58.34 | -38.79 | -97.13 |
| Control for **IP** | PGI_2_ | -54.01 | -42.39 | -96.40 |
| *^a^* All values are given in kcal·mol^−1^, obtained by the cDocker module (Discovery Studio 3.1);  *^b^* RSG = rosiglitazone, LRG= (2S)-2-(biphenyl-4-yloxy)-3-phenylpropanoic acid, PGI_2_ = prostacyclin;  *^c^*The average of **Full** (fully active form of PPARγ-LBD with coactivator peptide) and **Partial** (partially active form of PPARγ-LBD) groups. | | | | |
