# Supplementary material for: Cefminox, a Dual Agonist of Prostacyclin Receptor and Peroxisome Proliferator-Activated Receptor-Gamma Identified by Virtual Screening, Has Therapeutic Efficacy against Hypoxia-Induced Pulmonary Hypertension in Rats
Source: Front Pharmacol. 2018 Feb 23;9:134. doi: 10.3389/fphar.2018.00134 (PMC5829529; doi:10.3389/fphar.2018.00134)
Supplement: Supplementary file 2 [file Image1.PDF]

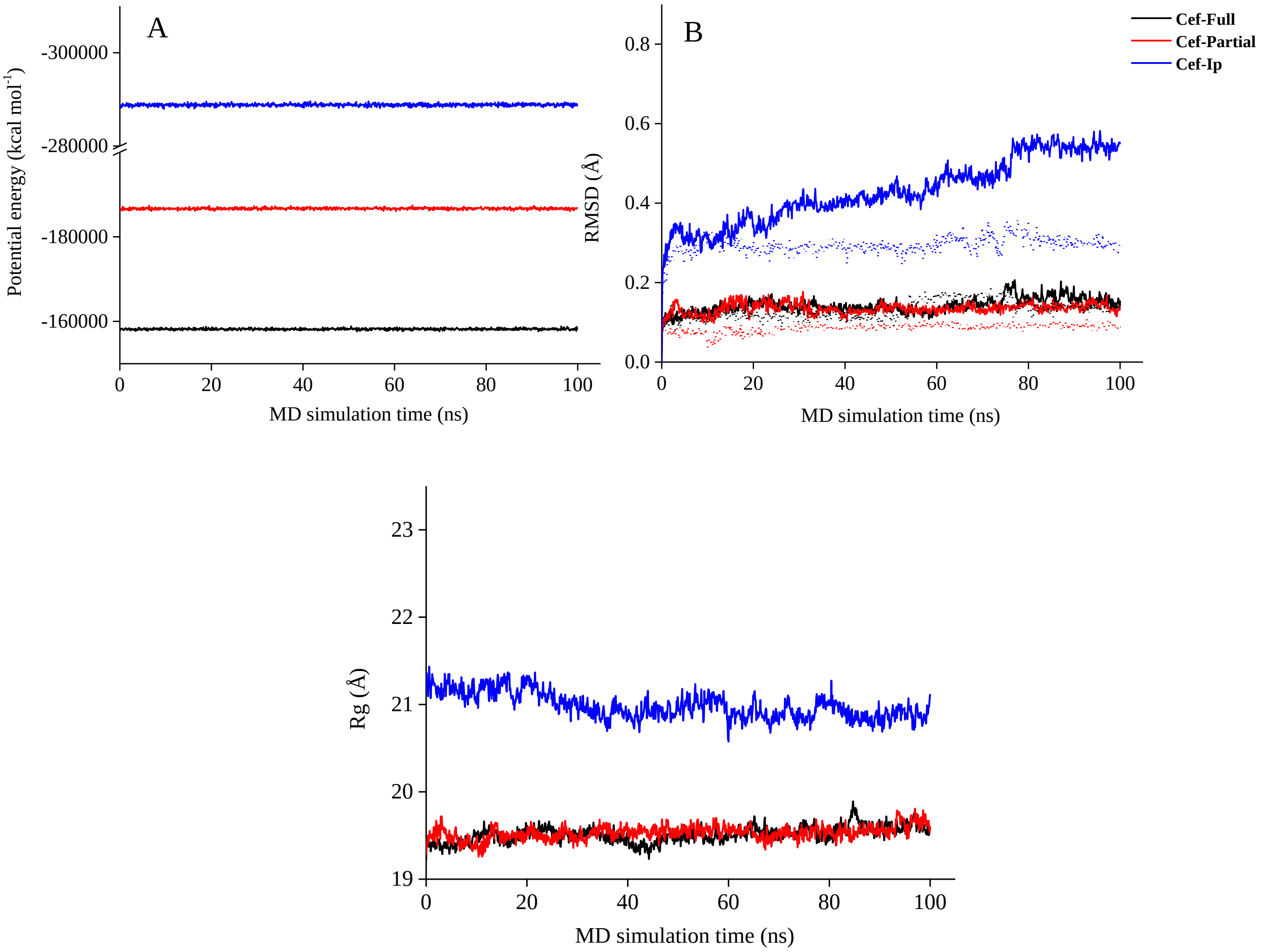

Figure S1. Variation of the potential energy, backbone-atom root-mean-square deviations (RMSD), and backbone radius of gyration (Rg) for various systems during 100 ns molecular dynamics (MD) simulation.

Ligand positional RMSDs are also represented by the dotted lines.
